# Emergence of *Neisseria meningitidis* Serogroup W, Central African Republic, 2015–2016

**DOI:** 10.3201/eid2411.170817

**Published:** 2018-11

**Authors:** Thierry Frank, Eva Hong, Jean-Robert Mbecko, Jean-Pierre Lombart, Muhamed-Kheir Taha, Pierre-Alain Rubbo

**Affiliations:** Institut Pasteur, Bangui, Central African Republic (T. Frank, J.-R. Mbecko, J.-P. Lombart, P.-A. Rubbo);; Institut Pasteur, Paris, France (E. Hong, M.-K. Taha)

**Keywords:** epidemics, serogroup W, meningitis/encephalitis, *Neisseria meningitidis*, Central African Republic, sub-Saharan Africa, meningococci, surveillance, bacteria

## Abstract

We analyzed data from the 2015 and 2016 meningitis epidemic seasons in Central African Republic as part of the national disease surveillance. Of 80 tested specimens, 66 belonged to meningococcal serogroup W. Further analysis found that 97.7% of 44 isolates belonged to the hyperinvasive clonal complex sequence type 11.

Central African Republic (CAR) is localized in the meningitis belt that spans from Senegal to Ethiopia in sub-Saharan Africa. The country experiences meningitis epidemics every year. Eight health districts (Nana Mambéré, Ouham Pendé, Ouham, Nana Gribizi, Bamingui Bangoran, Vakaga, Haute-Kotto, and Ouaka) are at particularly high risk of meningitis outbreaks during the dry season, November–March (Figure 1). During this period, epidemiologic surveillance is reinforced by the Health Ministry with the help of the World Health Organization (WHO), Institut Pasteur de Bangui (IPB; Bangui, CAR), and nongovernmental organizations, depending on the geographic zone.

**Figure 1:**
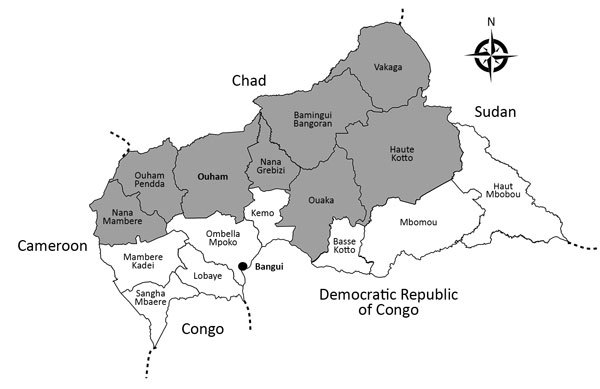
Health districts in Central African Republic. Gray regions correspond to those in the meningitis belt with higher risk for meningitis outbreaks each year. The names of Bangui Prefecture, where the main laboratory (Institut Pasteur) is located, and Ouham Prefecture, where all the 2015 and 2016 meningitis cases occurred, are in bold.

The gram-negative bacterium *Neisseria meningitidis* remains the leading cause of meningitis epidemics in Africa. Because of political troubles in the past years and missing reliable information available from the provinces, few epidemiologic and biologic data are available in CAR. Two previous reports, published by IPB in 2006 and 2008, had focused only on cases in Bangui (*1*,*2*). Data showed that, among proven bacterial disease, *Streptococcus pneumoniae* was the most common organism responsible for meningitis, resulting in a 47% death rate (29/62 cases). *N. meningitidis*, especially serogroup A (NmA), was also responsible for menigitis in Bangui to a lesser extent (*1*,*2*).

Historically, NmA of sequence types (STs) 5 and 7 was predominant in sub–Saharan Africa; serogroups W (ST11), C, and X (ST181) caused smaller numbers of cases. After introduction of massive vaccination campaigns, serogroup A incidence has massively decreased, and meningococcal strains causing disease shifted toward non-A serogroups (*3*). In particular, after the 1996–1997 epidemic in Africa that killed nearly 250,000 persons, the introduction of the MenAfriVac vaccine in 2010 contributed to dramatically reduced NmA cases in 26 countries of the meningitis belt by targeting roughly 65 million persons 1–29 years of age (WHO, http://www.who.int/immunization/newsroom/menafrivac_20121114/en/). Consequently, serogroup replacement has been noted. For example, in 2015, an epidemic with a novel strain of serogroup C was recorded in Niger and Nigeria for the first time since 1975 (WHO, http://www.who.int/wer/2015/wer9047.pdf?ua=1).

Hyperinvasive *N. meningitidis* serogroup W (NmW) cases belonging to the clonal complex ST11 (NmW/cc11) have been reported worldwide since the Hajj 2000–linked outbreak (*3*–*6*). Moreover, NmW/cc11 cases have been reported in the African meningitis belt at least since the late 1990s (*7*). Epidemics caused by these isolates were reported in 2001 and declined thereafter, but NmW/cc11 seems to have reemerged since 2010 (*8*). The genetic relationships between these NmW/cc11 isolates have been recently resolved using whole-genome sequencing, and 2 major NmW/cc11 lineages were described—the Anglo-French Hajj and South American/UK strains—in addition to several local NmW/cc11 isolates (*5*). We aimed to explore the genetic relationships of the 2015 and 2016 isolates from CAR with this reported population structure of NmW/cc11.

## The Study

This study was a collaborative work led by IPB. In 2015 and 2016, CAR experienced annual national meningitis epidemics. All cases confirmed positive for meningococcal meningitis in IPB were from 7 districts in Ouham Prefecture: Batangafo, Besse, Lady, Kambakota, Ouogo, Bongonon, and Kabo.

Each suspected patient meeting the recommended WHO case definition for meningitis (sudden onset of fever >38.5°C rectal or >38.0°C axillary and 1 of the following signs: neck stiffness, altered consciousness, or other meningeal sign) underwent a lumbar puncture and received antimicrobial drug therapy. In some cases, when available, a latex agglutination test (Pastorex; Bio-Rad, Hercules, CA, USA) was done on cerebrospinal fluid (CSF) samples before an aliquot and a clinical case report form for each patient were transported to IPB. We analyzed each sample according to procedures routinely used in IPB for diagnosis and monitoring of meningitis, according to WHO methodology (http://www.who.int/csr/resources/publications/meningitis/WHO_CDS_CSR_EDC_99_7_EN/en/). In brief, laboratory methodology used for NmW identification included Gram stain procedure, primary culture of CSF or transisolate inoculated medium onto blood agar plate and chocolate agar, and PCR testing. If CSF was visibly cloudy when received, we also performed latex agglutination (Pastorex) in the laboratory. NmW was considered as the causative agent when identified by culture, PCR, or both.

We performed molecular identification and genogroup determination using multiplex PCR (*9*,*10*) and multilocus sequence typing (MLST) using the methodology of the PubMLST *Neisseria* database (http://pubmlst.org/neisseria) on CSF samples (n = 44) using a single round of PCR. We subjected a proportion of cultured isolates (n = 28) to whole-genome sequencing using Illumina NextSeq 500 (Illumina, San Diego, CA, USA) and assembled them as described previously (*11*). We compared genomes using the PubMLST Genome Comparator tool on the basis of core genome (*12*). We visualized phylogenetic trees using SplitsTree4 version 4.13.1 (http://www.splitstree.org).

In 2015 and 2016, a total of 276 suspected cases of meningitis were reported in CAR (Table). Among these 276 patients, 13 died in 2015 and 12 in 2016. Of the 80 CSF samples received at IPB for biologic investigations, 52 bacteriologic cultures and 66 PCR tests were positive for NmW. All samples that were positive in culture were also positive in PCR. All 66 positive PCR tests identified serogroup W, confirming the presence of such epidemic strains in central Africa. We also found 5 PCR tests positive for *Streptococcus pneumoniae*, as well as 9 negative results for both culture and PCR assays on the CSF samples. The most common signs and symptoms among the confirmed NmW case-patients were fever (97%), weakness (85%), and neck stiffness (78%).

**Table:** Details of suspected and confirmed meningitis cases caused by *Neisseria meningitidis* during the 2015 and 2016 outbreaks in Central African Republic*

Data	2015	2016
Demographics		
Suspected cases, no.	120	156
CSF samples tested, no. (%)	20 (17)	60 (39)
Median age (IQR), mo	60 (36–162)	66 (21–186)
Sex ratio, M/F	11/9	36/24
Clinical data, no. (%)		
Fever	ND†	58 (97)
Weakness/asthenia	ND†	51 (85)
Neck stiffness	ND†	47 (78)
Deaths	13 (11)	12 (8)
Laboratory results, no. (% samples received)		
Positive NmW culture	11 (55)	41 (68)
Positive NmW PCR	20 (100)	46 (77)

*CSF, cerebrospinal fluid; IQR, interquartile range; ND, not determined; NmW, *N. meningitidis* serogroup W. †Clinical information is missing for suspected cases during the 2015 epidemics.

**Figure 2:**
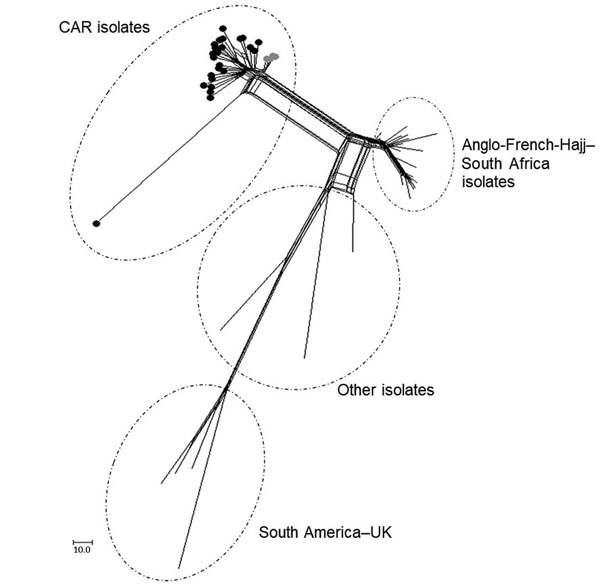
Core genome multilocus sequence typing (MLST) neighbor-net phylogenetic network for the temporal distribution of *Neisseria meningitidis* serogroup W clonal complex sequence type cc11 isolates from CAR (n = 28) and reference isolates. The tree was built with neighbor-net SplitsTree graphs generated by SplitsTree4 version 4.13.1 (http://www.splitstree.org). The lineages Anglo-French-Hajj and South American/UK are indicated; other indicates 13 isolates retrieved from or based on the work of Lucidarme et al. (*5*). The isolates from CAR are also indicated by gray circles for the isolates of 2015 (n = 4 corresponding to on the PubMLST *Neisseria* database [http://pubmlst.org/Neisseria], 41187–41191) and by black circles for the isolates of 2016 (n = 24 corresponding to the PubMLST database, 44004–44027). The 30 isolates from the PubMLST database are as follows: 2290 (Saudi Arabia 2000); 19957 (United Kingdom 2000); 27087 (Burkina Faso 2002); 21588, 29326, 29337, 29370, 29371, 29382, 29384, 29401, 29406, 29422 (South Africa 2004–2005); 29775, 29928, 29929 (United Kingdom 2000); 30065, 30066, 30067 (Turkey 2005–2006); 30074 (Algeria 1999); 30075, 30079, 30107 (Senegal 2000–2001); and 31164, 31165, 42409, 42758, 42767, 42769, 50815 (France 2000–2016). CAR, Central African Republic.

From the 66 confirmed NmW cases, 44 CSF samples were sent to the WHO collaborating reference center for meningitis located in Institut Pasteur, Paris, for diagnostic confirmation and genetic analyses. Additionally, 28 isolates underwent genome sequence analysis for evaluating the genetic relationships of NmW strains identified in CAR to others during epidemics elsewhere. Phylogeny results clearly indicated that all the CAR isolates that underwent next-generation sequencing (4 in 2015 and 24 in 2016) were genetically linked. These isolates grouped together in a genetic cluster separated from other NmW/cc11 strains previously described, including the Anglo-French Hajj branch and the South American/UK branch, and from other local strains found in Africa (Figure 2).

Compared with data obtained in Bangui in the past years, which highlighted NmA as the main etiology of meningitis cases, our data shows that the strains isolated during epidemic seasons in the north of CAR in 2015 and 2016 were all NmW. In addition, most of the characterized cases (43/44) belonged to the ST11 complex and had the antigenic formula NmW:P1.5,2:F1–1:cc11. The other case isolated in 2016 (1/44) belonged to the ST175 complex, a nonhyperinvasive clonal complex that should promote immune exploration, as such isolates have been reported more frequently among patients with complement deficiencies (*13*).

## Conclusions

The number of meningitis cases reported during the 2015 and 2016 epidemic seasons was the most documented in CAR since 2008. We described the presence of a specific endemic NmW strain that appears to be circulating in sub–Saharan Africa, including in CAR. Foci correspond to endemic local strains, which confirms the hypothesis of the multifocal emergence of specific NmW/cc11 strains (*5*,*14*,*15*).

Although CAR experiences epidemics of meningitis each year, the weaknesses of the national health system contribute to a delay in management of suspected cases. The emergence or reemergence of epidemic NmW strains in CAR requires persistent public health surveillance and increased capacity of epidemic detection and prevention, as well as revised vaccination policy.
